# Supplementary material for: Land tenure regimes influenced long-term restoration gains and reversals across Brazil’s Atlantic forest
Source: Nat Commun. 2025 Oct 31;16:9656. doi: 10.1038/s41467-025-64732-0 (PMC12578834; doi:10.1038/s41467-025-64732-0)
Supplement: Supplementary file 1 — Supplementary Information [file 41467_2025_64732_MOESM1_ESM.pdf]

Supplementary Information

Description of land tenure regime transitions

We do not believe that many lands transitioned between land tenure regimes during the study period, which corresponds to the claims of another similar study (Pacheco & Meyer, 2022). For example, most private properties (PPs) had already been recognized well before the enactment of the Brazilian Constitution in 1988 (Pacheco & Meyer, 2022), and before the start of our study period in 1985. Those PPs that shifted ownership during the study period did not influence the broader land tenure regime, and therefore did not influence our results. In addition, communities living in Indigenous lands (ILs) and *Quilombola* territories (QTs) have ancestral ties to territory, and were most likely residing in their same territories before these territories were legally recognized. Protected areas (PAs) and agrarian-reform settlements (ARSs) were primarily established from lands that were previously public lands (PLs). PAs were primarily established before the start of our study period, and ARSs were primarily established during the first fifteen years of the study period (although settlers often arrived before the date of legal establishment).

Imaflora land tenure regime classifications

We merged several of the more specific Imaflora land regime classifications to represent the broader classifications of PPs, ILs, QTs, ARSs, PAs, and PLs (De Freitas et al., 2018). PPs encompassed the Imaflora classifications of "SIGEF", "CAR premium", and "CAR poor" (to include PPs from different databases managed by different government bodies, and of differing degrees of overlap with other existing properties); ILs encompassed the sub-classifications of "Terra Indígena homologada" and "Terra Indígena não homologada" (to include both tenured and non-tenured ILs); QTs and ASs each only had one sub-classification by Imaflora; PAs encompassed "UC Proteção Integral" and "UC Uso Sustentável" (see Table 1 of main manuscript); and PLs encompassed "SIGEF SNCI público" and "Florestas Tipo B" (public properties and undesignated public forests). See Imaflora documentation for the detailed definitions of each sub-classification.

We combined both types of ILs into one category due to the precedence from a previous study (Pacheco & Meyer, 2022), which also assessed differences between land tenure regimes rather than differences in land tenure formalization. Similarly, we included both types of PAs into one category to test the relationship between PAs and other land tenure regimes broadly, rather than testing subclassifications unique to Brazil's jurisdictional land classifications specifically. Although some past studies have tested ILs and QTs in combination (e.g., Alves-Pinto et al., 2022), we tested them separately to tease out unique trends for each land tenure regime. However, our model of ILs and QTs tested together also had significant results (Table S1). Similar to other studies (Pacheco & Meyer, 2022), we did not include Imaflora's land tenure regime "Territórios Comunitários" (communal territories), due to inadequate sample size (n=12), and did not include military lands, urban areas, water bodies, or transportation network lands in the analysis. The Imaflora dataset is preprocessed and thoroughly cleaned to remove duplicates, self-overlaps, overlaps between databases (with priority given to most recent data), and properties that lost more than 50% of their area after overlaps. Past studies have claimed that the Imaflora dataset likely represents "the most reliable and comprehensive parcel-level land-tenure information available for any large tropical country" (Pacheco & Meyer, 2022).

Model results

Table S1. Estimates and standard errors of regression models after matching for restoration reversals and long-term restoration gains. All models are matched with private properties. Effect sizes were fit based on treatment condition and cannot be compared between models.

Model	Effect: *Long-term restoration gain*	Standard error: *Long-term restoration gain*	Effect: *Restoration reversal*	Standard error: *Restoration reversal*
Indigenous lands	189***	45.2 (Z=4.19; p<0.001)	21.0*	9.62 (Z=2.18; p=0.029)
Agrarian-reform settlements	6.09*	3.07 (Z=1.98; p=0.047)	4.66***	0.77 (Z=6.09; p<0.001)
Quilombola territories	7.76	13.4 (Z=0.577; p=0.56)	1.28	3.13 (Z=0.408; p=0.68)
Protected areas	-30.2	55.9 (Z=-0.541; p=0.59)	-0.28	7.98 (Z=-0.035; p=0.97)

** p < 0.05, ** p < 0.01, *** p < 0.001*

Model covariates

Table S2. Model covariates, definitions and units, source, and year released. IBGE represents the Brazilian Institute of Geography and Statistics. SRTM represents Shuttle Radar Topography Mission.

**Variable**	**Definition and units**	**Source (and year of citation based on data access)**	**Year (dataset released)**
Territory size	Total area of territory (ha)	Imaflora (Freitas et al., 2018)	2018
Percentage of forest cover in 1985	Amount of forest cover in territory in 1985 divided by territory size times 100 (%)	MapBiomas (2023)	2022
Temperature	Mean annual temperature 1970-2000 (celsius)	WorldClim (Version 2.1) (Fick & Hijmans, 2017)	2020
Precipitation	Mean annual precipitation 1970-2000 (mm)	WorldClim (Version 2.1) (Fick & Hijmans, 2017)	2020
Slope	Mean slope (degrees)	SRTM Digital Elevation Data (Version 4) (Jarvis et al., 2008)	2008
Elevation	Mean elevation (m)	SRTM Digital Elevation Data (Version 4) (Jarvis et al., 2008)	2008

Distance to roads	Mean distance to roads (m)	IBGE road and hydrography layer (2019)	2016
Distance to rivers	Mean distance to rivers (m)	IBGE road and hydrography layer (2019)	2016
Distance to nearest city	Travel time to nearest city of 50,000 or more people in 2000 (minutes)	Nelson (2008)	2008
Population density	Mean population density (pop/km^2)	IBGE Demographic Census (2019)	2016

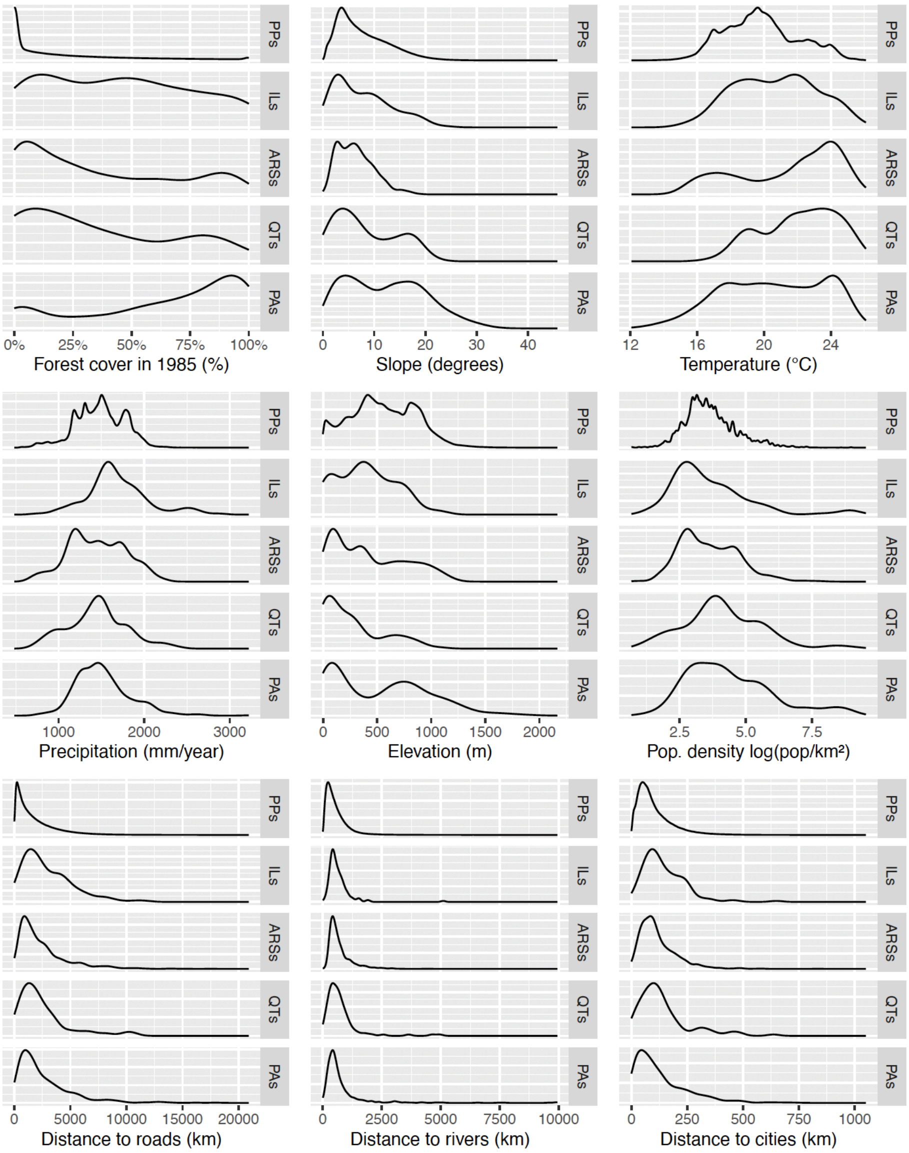

Figure S1. Distributions of covariate variation by land tenure regime.

Model robustness

Table S3. Robustness checks. Model results are robust to a variety of ways of matching, including with and without optimal full matching (OFM) and with and without discarding units outside the region of common support (dropping less well-balanced matches).

Model (vs. private properties (PPs))	Restoration outcome	Optimal full matching	Dropped	Average estimate	Standard deviation	Proportion significant
Indigenous lands (ILs)	Long-term gain	FALSE	FALSE	190.62	6.30	1.00
		FALSE	TRUE	190.62	6.30	1.00
		TRUE	FALSE	190.62	6.30	1.00
		TRUE	TRUE	193.93	5.88	1.00
	Reversal	FALSE	FALSE	21.12	0.86	1.00
		FALSE	TRUE	21.12	0.86	1.00
		TRUE	FALSE	21.12	0.86	0.98
		TRUE	TRUE	20.89	0.79	1.00
Agrarian-reform settlements (ARSs)	Long-term gain	FALSE	FALSE	6.21	0.26	1.00
		FALSE	TRUE	6.21	0.26	1.00
		TRUE	FALSE	6.21	0.26	0.91
		TRUE	TRUE	6.13	0.26	0.82
	Reversal	FALSE	FALSE	4.57	0.12	1.00
		FALSE	TRUE	4.57	0.12	1.00
		TRUE	FALSE	4.57	0.12	1.00
		TRUE	TRUE	4.53	0.12	1.00
Quilombola territories (QTs)	Long-term gain	FALSE	FALSE	4.30	4.30	0.00
		FALSE	TRUE	4.30	4.30	0.00
		TRUE	FALSE	4.30	4.30	0.00
		TRUE	TRUE	0.84	4.69	0.00
	Reversal	FALSE	FALSE	-0.16	1.31	0.00
		FALSE	TRUE	-0.16	1.31	0.00
		TRUE	FALSE	-0.16	1.31	0.00
		TRUE	TRUE	0.30	1.17	0.00
Protected areas (PAs)	Long-term gain	FALSE	FALSE	-26.29	3.09	0.00
		FALSE	TRUE	-26.29	3.09	0.00

		TRUE	FALSE	-26.29	3.09	0.00
		TRUE	TRUE	-26.52	3.10	0.00
	Reversal	FALSE	FALSE	-0.36	0.58	0.00
		FALSE	TRUE	-0.36	0.58	0.00
		TRUE	FALSE	-0.36	0.58	0.00
		TRUE	TRUE	-0.37	0.58	0.00
Public lands (PLs)	Long-term gain	FALSE	FALSE	0.66	0.00	0.00
		FALSE	TRUE	0.66	0.00	0.00
		TRUE	FALSE	0.66	0.00	0.00
		TRUE	TRUE	0.69	0.00	0.00
	Reversal	FALSE	FALSE	-0.17	0.00	1.00
		FALSE	TRUE	-0.17	0.00	1.00
		TRUE	FALSE	-0.17	0.00	1.00
		TRUE	TRUE	-0.16	0.00	1.00
ILs and QTs (combined)	Long-term gain	FALSE	FALSE	85.44	7.14	1.00
		FALSE	TRUE	85.44	7.14	1.00
		TRUE	FALSE	85.44	7.14	1.00
		TRUE	TRUE	89.98	6.83	1.00
	Reversal	FALSE	FALSE	8.22	1.50	0.43
		FALSE	TRUE	8.22	1.50	0.43
		TRUE	FALSE	8.22	1.50	0.35
		TRUE	TRUE	8.51	1.38	0.43

Visual diagnostics and alternate model comparisons

We developed the agglomerative matching approach to achieve adequate covariate balance when matching treatment lands and PPs without dropping data. We first ran non-agglomerative matching. We selected the approach of generalized full matching due to the large sample size of our dataset; there are 1.88 million private properties compared to 1,408 ARSs and 143 ILs. Conducting matching without dropping data requires a more scalable method than OFM. We found that we could not achieve adequate balance using generalized full matching alone (Fig. S1).

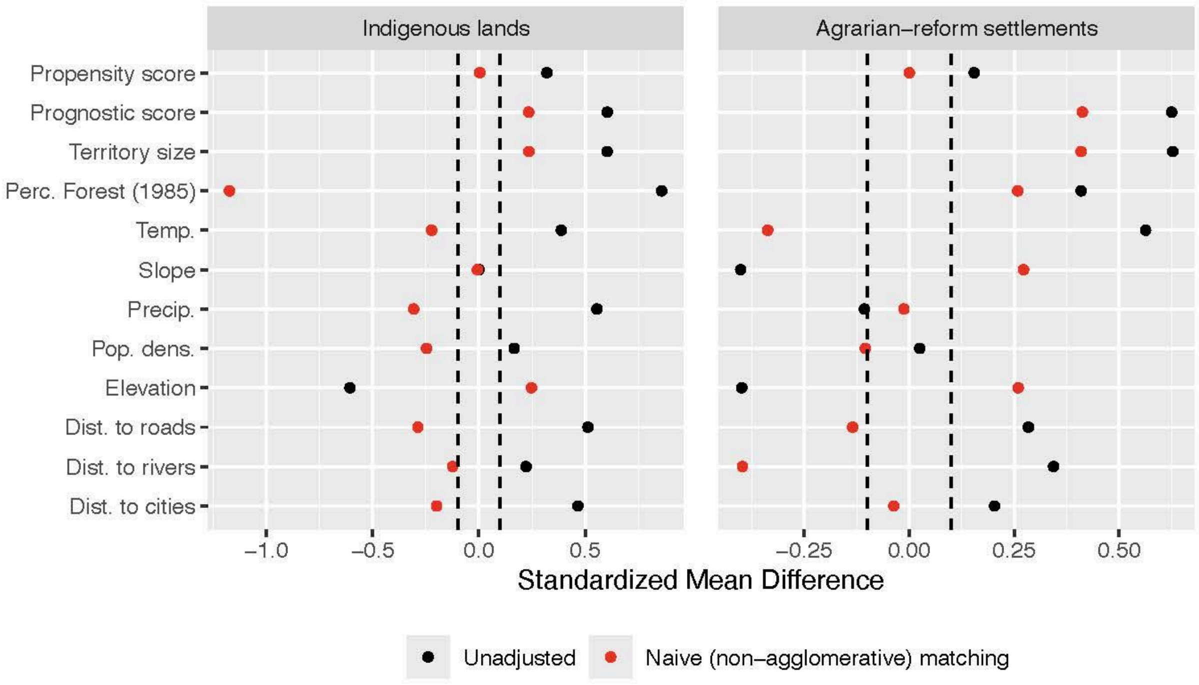

Figure S2. Non-agglomerative matching did not result in adequate balance. Plots of covariate balance before and after generalized full matching between land tenure regimes and all non-agglomerated private properties, as well as the propensity and prognostic scores. Dashed vertical lines at -0.1 and 0.1 represent common thresholds of acceptable balance. Standardized mean difference of each covariate is reported for each condition before and after matching. Many covariates remained imbalanced even after matching.

An alternative approach could have been to select a matching method that allows treatment units to be dropped, such as coarsened exact matching, and to reweight the remaining data to offset the missing data (e.g., Pacheco & Meyer, 2022). Such an approach allows for better covariate balance in exchange for dropping data. However, we have few treatment lands in both relative and absolute terms, and the characteristics of the treatment lands that would be dropped may be systematically different from the non-dropped treatment lands. For example, ILs that are far larger than any corresponding PPs would likely have been dropped, and yet may be different in important ways from smaller ILs that could not have been properly accounted for by reweighting the other covariates.

Agglomerative matching resolved this issue by assuming that agglomerates of PPs exhibited similar characteristics as a corresponding larger PP with the same covariates. We tested for the robustness of our results to specific individual agglomerations by rerunning agglomerative matching using different random seeds for the approximate nearest neighbors index, which generated slightly different combinations of agglomerations. To ensure that all covariates were properly balanced after running the agglomeration algorithm, we ran a second round of OFM between the PPs (control) and the agglomerated lands (treatment), which led to overall better balance (Fig. S2). Without this step some covariates remained imbalanced. However, results were similar regardless of whether this second rebalancing occurred or not (Table S2). The PLs facet (Fig. S2) demonstrates that balance was not sufficient enough to include PL models in the analysis.

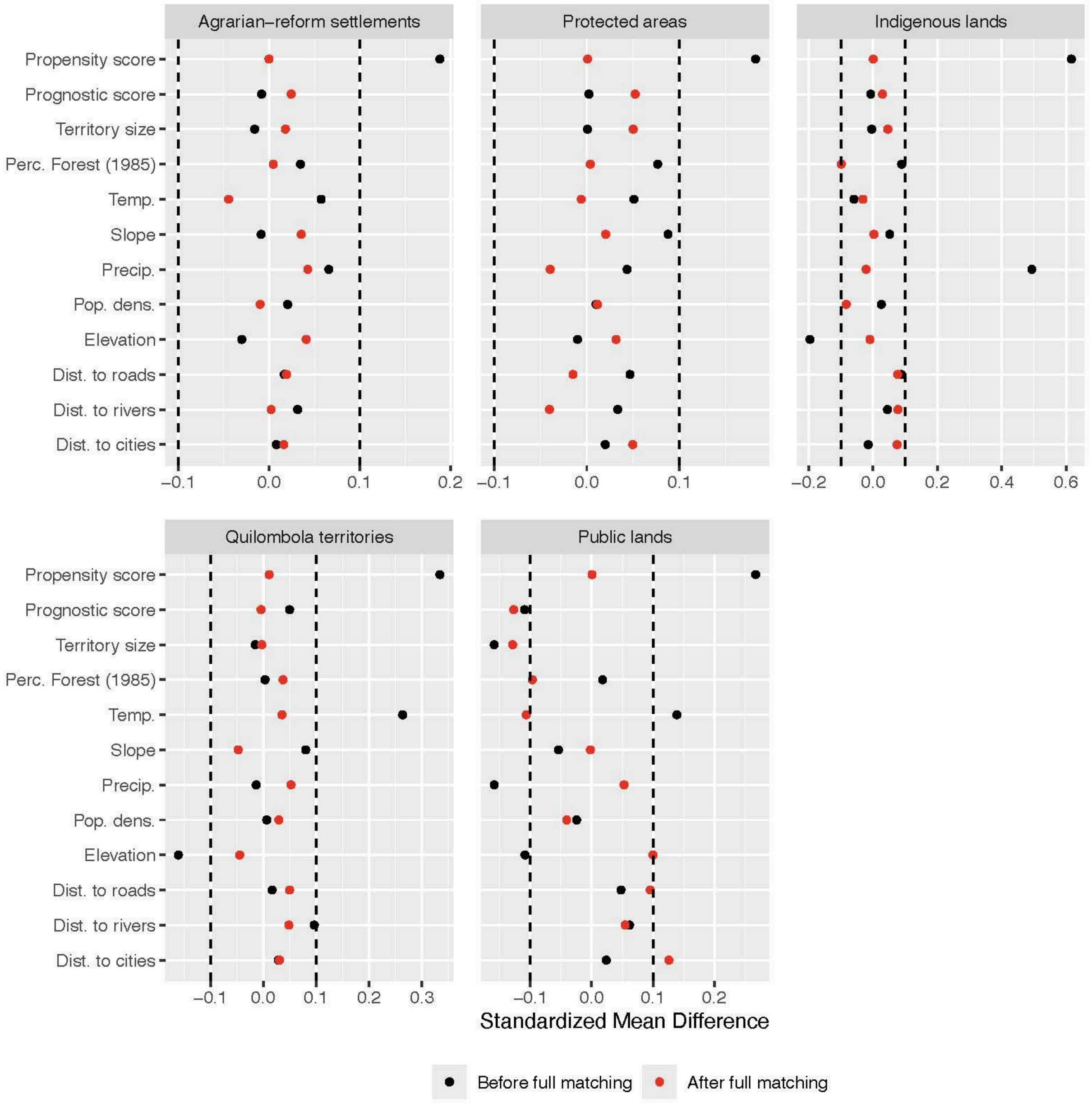

Figure S3. Covariate balance before and after optimal full matching (OFM) and following agglomeration. Each facet includes a land tenure regime that is compared to agglomerated private properties, including the propensity and prognostic scores. Dashed vertical lines at -0.1 and 0.1 represent common thresholds of acceptable balance. For each condition, we display the most balanced set of covariates out of 500 different random seeds, where most balanced is defined as having the minimum maximum absolute standardized mean difference across the covariates. All conditions fall within the threshold of [-0.1, 0.1] after full matching except public lands, which were omitted from the analysis.

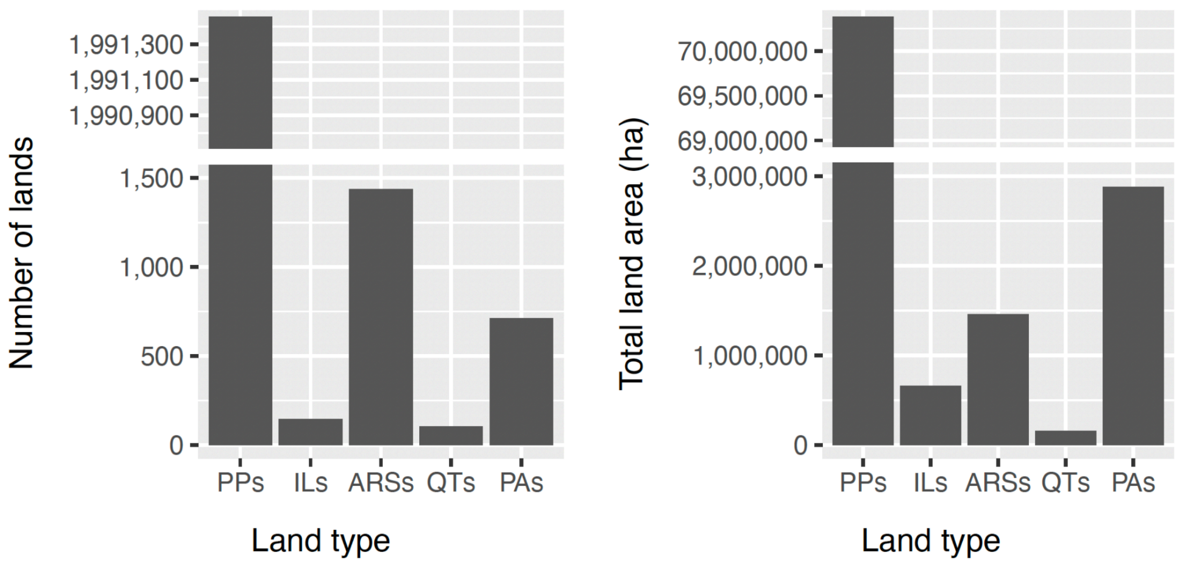

Figure S4. Number of lands and total land area of land tenure regimes.

Limitations of agglomerative matching

One assumption of our analysis is that there are no additional boundary effects due to the increased number of borders in private agglomerates. For example, borders could prevent restoration when landowners deforest land that regenerated from other nearby properties, or borders could increase restoration when cattle is fenced off and prevented from grazing nearby forests. Nevertheless, we did not believe that this factor was politically relevant. Given that there is no policy proposal to combine the borders of PPs into larger lands, we measured restoration effects in PPs using the borders that currently exist. Alternative approaches, such as measuring restoration as a percentage would have been less politically-relevant than our current approach, given that this technique highly weights tiny properties that have large percentages of long-term restoration gains, rather than giving a higher weight to properties with large areas of land restoration.

Supplementary references

1. Alves-Pinto, H. N., L.O. Cordeiro, C., Geldmann, J., D. Jonas, H., Gaiarsa, M. P., Balmford, A., E.M. Watson, J., Latawiec, A. E., & Strassburg, B. (2022). The role of different governance regimes in reducing native vegetation conversion and promoting regrowth in the Brazilian Amazon. *Biological Conservation*, *267*, 109473. https://doi.org/10.1016/j.biocon.2022.109473
2. Fick, S.E. and Hijmans, R.J. (2017). WorldClim 2: new 1km spatial resolution climate surfaces for global land areas. *International Journal of Climatology* 37 (12): 4302-4315.
3. De Freitas, F.L.M., Guidotti,V., Sparovek, G. & Hamamura, C. (2018). Nota técnica: Malha fundiária do Brasil. *Atlas–A Geografia da Agropecuária Brasileira; IMAFLORA: Piracicaba, Brazil, 1812*, 5. Available at: www.imaflora.org/atlasagropecuario
4. Instituto Brasileiro de Geografia e Estatística (IBGE) (2019). Sistema IBGE de Recuperação Automática — SIDRA. Available at: https://sidra.ibge.gov.br/acervo

5. Jarvis, A., Reuter, H.I., Nelson, A., Guevara, E. (2008). Hole-filled SRTM for the globe Version 4, available from the CGIAR-CSI SRTM 90m Database: https://srtm.csi.cgiar.org
6. Pacheco, A., & Meyer, C. (2021). *Land-tenure regimes determine tropical deforestation rates across socio-environmental contexts*.
7. MapBiomas Project - Collection 8 of the Annual Land Use Land Cover Maps of Brazil, accessed on November 8th 2023 through the link: https://brasil.mapbiomas.org/
