# Supplementary material for: Land tenure regimes influenced long-term restoration gains and reversals across Brazil’s Atlantic forest
Source: Nat Commun. 2025 Oct 31;16:9656. doi: 10.1038/s41467-025-64732-0 (PMC12578834; doi:10.1038/s41467-025-64732-0)
Supplement: Supplementary file 2 — Reporting Summary [file 41467_2025_64732_MOESM2_ESM.pdf]

nature portfolio

Corresponding author(s): DAPR

Last updated by author(s): Apr 25, 2025

Reporting Summary

Nature Portfolio wishes to improve the reproducibility of the work that we publish. This form provides structure for consistency and transparency in reporting. For further information on Nature Portfolio policies, see our Editorial Policies and the Editorial Policy Checklist.

Statistics

For all statistical analyses, confirm that the following items are present in the figure legend, table legend, main text, or Methods section.

n/a	Confirmed	
☐	☒	The exact sample size (n) for each experimental group/condition, given as a discrete number and unit of measurement
☐	☒	A statement on whether measurements were taken from distinct samples or whether the same sample was measured repeatedly
☐	☒	The statistical test(s) used AND whether they are one- or two-sided<br>*Only common tests should be described solely by name; describe more complex techniques in the Methods section.*
☐	☒	A description of all covariates tested
☐	☒	A description of any assumptions or corrections, such as tests of normality and adjustment for multiple comparisons
☐	☒	A full description of the statistical parameters including central tendency (e.g. means) or other basic estimates (e.g. regression coefficient) AND variation (e.g. standard deviation) or associated estimates of uncertainty (e.g. confidence intervals)
☐	☒	For null hypothesis testing, the test statistic (e.g. F, t, r) with confidence intervals, effect sizes, degrees of freedom and P value noted<br>*Give P values as exact values whenever suitable.*
☒	☐	For Bayesian analysis, information on the choice of priors and Markov chain Monte Carlo settings
☒	☐	For hierarchical and complex designs, identification of the appropriate level for tests and full reporting of outcomes
☐	☒	Estimates of effect sizes (e.g. Cohen's d, Pearson's r), indicating how they were calculated

Our web collection on statistics for biologists contains articles on many of the points above.

Software and code

Policy information about availability of computer code

Data collection	Please see data availability statement
Data analysis	Please see data availability statement

For manuscripts utilizing custom algorithms or software that are central to the research but not yet described in published literature, software must be made available to editors and reviewers. We strongly encourage code deposition in a community repository (e.g. GitHub). See the Nature Portfolio guidelines for submitting code & software for further information.

Data

Policy information about availability of data

All manuscripts must include a data availability statement. This statement should provide the following information, where applicable:

- Accession codes, unique identifiers, or web links for publicly available datasets
- A description of any restrictions on data availability
- For clinical datasets or third party data, please ensure that the statement adheres to our policy

Our dataset was assembled from publicly and openly available sources. Code to reproduce the model results is hosted at: https://github.com/samzhang111/agglomerative-matching-reforestation

Research involving human participants, their data, or biological material

Policy information about studies with human participants or human data. See also policy information about sex, gender (identity/presentation), and sexual orientation and race, ethnicity and racism.

Reporting on sex and gender	N/A
Reporting on race, ethnicity, or other socially relevant groupings	Data was analyzed on Indigenous lands, Quilombola territories, and agrarian-reform settlements. These lands are legal territorial designations that represent certain socially-relevant groupings. These groupings were used to test forest restoration outcomes between different land tenure regimes.
Population characteristics	One covariate used in the analysis was 'population density', which represented the approximate density of population per territory.
Recruitment	N/A
Ethics oversight	N/A

Note that full information on the approval of the study protocol must also be provided in the manuscript.

Field-specific reporting

Please select the one below that is the best fit for your research. If you are not sure, read the appropriate sections before making your selection.

☐ Life sciences ☒ Behavioural & social sciences ☐ Ecological, evolutionary & environmental sciences

For a reference copy of the document with all sections, see nature.com/documents/nr-reporting-summary-flat.pdf

Behavioural & social sciences study design

All studies must disclose on these points even when the disclosure is negative.

Study description	Our study was a quantitative analysis. Focusing on restoration reversals—restored forests that were later deforested—and restoration successes—restored forests that remained forested, our study investigates the longevity of forest restoration across 1.9 million territories in the Atlantic Forest of Brazil from 1985 to 2022. We conduct a causal analysis in Indigenous lands, Afro-descendant (Quilombola) territories, agrarian-reform settlements (assentamentos), and protected areas in comparison to private properties.
Research sample	Our sample included all territories that were available from our dataset in the Atlantic Forest biome of Brazil.
Sampling strategy	N/A
Data collection	Data was sourced from the Atlas da Agropecuária Brasileira from Imaflora and Collection 8 of the MapBiomas Project.
Timing	Data was downloaded between March 2023 and April 2024.
Data exclusions	We included all data for the territories we measured from the two datasets.
Non-participation	N/A
Randomization	N/A

Reporting for specific materials, systems and methods

We require information from authors about some types of materials, experimental systems and methods used in many studies. Here, indicate whether each material, system or method listed is relevant to your study. If you are not sure if a list item applies to your research, read the appropriate section before selecting a response.

Materials & experimental systems

n/a	Involved in the study
☒	☐ Antibodies
☒	☐ Eukaryotic cell lines
☒	☐ Palaeontology and archaeology
☒	☐ Animals and other organisms
☒	☐ Clinical data
☒	☐ Dual use research of concern
☒	☐ Plants

Methods

n/a	Involved in the study
☒	☐ ChIP-seq
☒	☐ Flow cytometry
☒	☐ MRI-based neuroimaging

Plants

Seed stocks	N/A
Novel plant genotypes	N/A
Authentication	N/A
